# Fish Pluripotent Stem-Like Cell Line Induced by Small-Molecule Compounds From Caudal Fin and its Developmental Potentiality

**DOI:** 10.3389/fcell.2021.817779

**Published:** 2022-01-20

**Authors:** Wenting Xu, Huajin Li, Liangyue Peng, Liyu Pu, Sijia Xiang, Yue Li, Leiting Tao, Wenbin Liu, Jinhui Liu, Yamei Xiao, Shaojun Liu

**Affiliations:** ^1^ State Key Laboratory of Developmental Biology of Freshwater Fish, Hunan Normal University, Changsha, China; ^2^ College of Life Sciences, Hunan Normal University, Changsha, China

**Keywords:** induced pluripotent stem cell, chemical reprogramming, fish, germ cell, small molecule compounds

## Abstract

The technique of induced pluripotent stem cells has significant application value in breeding and preserving the genetic integrity of fish species. However, it is still unclear whether the chemically induced pluripotent stem cells can be induced from non-mammalian cells or not. In this article, we first verify that fibroblasts of fish can be chemically reprogrammed into pluripotent stem cells. These induced pluripotent stem-like cells possess features of colony morphology, expression of pluripotent marker genes, formation of embryoid bodies, teratoma formation, and the potential to differentiate into germ cell-like cells *in vitro*. Our findings will offer a new way to generate induced pluripotent stem cells in teleost fish and a unique opportunity to breed commercial fish and even save endangered fish species.

## Introduction

Generation of induced pluripotent stem cells (iPSCs) can provide important insights into cell fate, mammalian development, and human diseases, and has found wide applications in the fields of animal cloning, production of transgenic animals, rescue and protection of animal genetic resources, the establishment of animal breeding technology system (Ben-Nun et al., 2011; Kumar et al., 2015; Omole and Fakoya, 2018; Pessoa et al., 2019; Rowe and Daley, 2019; Stanton et al., 2019; Scarfone et al., 2020). Traditional reprogramming methods used to produce iPS cells with genetic material included viral vectors (Takahashi and Yamanaka, 2006; Okita et al., 2007; Woltjen et al., 2009), which inevitably brought security risks to the application of iPS cells (Miura et al., 2009). With the development of reprogramming technology, a variety of small molecular compounds have been used to improve the efficiency of iPS induction (Zhang et al., 2012; Masuda et al., 2013). The targets of small molecular compounds are relatively clear, and they have rapid and reversible effects on the activation and inhibition of specific protein functions (Li et al., 2012). In 2013, Hou et al. successfully induced mouse somatic reprogramming using a pure chemical method, an essential step towards a safe reprogramming method (Hou et al., 2013). Subsequently, Deng and his colleagues found that small molecular compound-induced reprogramming was different from traditional gene-induced reprogramming in molecular regulatory pathways. Through the accurate use of the key steps in the process of compound-induced reprogramming, a new method to complete reprogramming with high efficiency was found (Zhao et al., 2015). In addition, Ye et al. demonstrated that the strategy of chemically induced pluripotent stem cells was repeatable in different donor cell types (Ye et al., 2016). Chemical reprogramming has unique advantages different from traditional reprogramming methods, and it also plays a great role in promoting the study of somatic reprogramming mechanisms (Zhao et al., 2015; Zhao et al., 2018; Zhao, 2019; Zhou et al., 2020a; Zhou et al., 2020b; Ma et al., 2021).

However, in the past decade, the iPSC technology mainly focused on mammalian species, such as mice, human and domestic animals (Takahashi and Yamanaka, 2006; Lowry et al., 2008; Shi et al., 2008; Esteban et al., 2009; Kim et al., 2009; Li et al., 2009; Liao et al., 2009; Wu et al., 2009; Honda et al., 2010; Shimada et al., 2010; Tancos et al., 2017; Sakai et al., 2019). Few attempts have been made in non-mammals (Rossello et al., 2013; Fuet and Pain, 2017; Peng et al., 2019). Compared with other vertebrates, fish species have the advantages of high spawning rate, transparent embryos, and rapid embryonic development, which have important application value in stem cell research, disease model, drug discovery, and other fields (Alvarez et al., 2007; Yan et al., 2009; Lin et al., 2016; Xiao et al., 2016; Chen et al., 2017). The iPS cells also have considerable applications in the field of non-mammalian cell research, such as the production of transgenic animals, the improvement of cloning efficiency, and the protection of wild animals (Ben-Nun et al., 2011; Pessoa et al., 2019; Stanton et al., 2019). This article selected Kio (*Cyprinus carpio haematopterus*) fibroblasts to verify the feasibility of pure chemical reprogramming in non-mammals. To optimize the pure chemical approach, we intended to investigate a pure chemical reprogramming method to induce fish cells and generate iPS-like cells from Kio fibroblasts. The results obtained in this experiment are more conducive to the wide application of iPS in non-mammal species.

## Materials and Methods

### Primary Cell Culture

The caudal fin fibroblasts of Kio (kFFs) were cultured as described previously (Peng et al., 2019). Briefly, fibroblasts were cultured in fibroblast medium, which was composed of Dulbecco’s modified Eagle’s medium (DMEM) supplemented with 100 U/mL penicillin (Invitrogen), 100 μg/ml streptomycin (Invitrogen), 10% fetal bovine serum (FBS, Invitrogen), 2.5% fish serum (Common Carp), 0.1% 2-mercaptoethanol (2-ME, Invitrogen), 1 mM sodium pyruvate (Invitrogen), and 1 mM nonessential amino acids (Invitrogen). The cells were cultured in 2.5% (v/v) CO_2_ at 28°C, and passage every 2 or 3 days.

### Pre-Experiment of CiPSLCs Induction From Fish Fibroblasts

To test whether pure chemical reprogramming could be applied in cells from fish species or not, we initially used the reported chemical induction protocol in mice to treat the kFFs (Zhao et al., 2015). The source of each small molecule compound is described in Supplementary materials (Supplementary Table S1). We found that the original chemical reprogramming cocktail “VC6TFZE” (V, VPA; C, CHIR 99021; 6, 616452; T, tranylcypromine; F, forskolin; Z, DZNep; E, EPZ004777) could not induce the pluripotency of kFFs, and a large number of cells lost their vitality after 10 days of treatment (Supplementary Figure S1B). After reducing the concentration of small molecules in chemical reprogramming cocktails to 50 and 25% of the original concentration, dense epithelial colonies were observed (Supplementary Figure S1C,D). However, when reducing the concentration of small molecules in chemical reprogramming cocktails to 20% of the original concentration, no epithelial colony emerged (Supplementary Figure S1E). In addition, after switching to the third stage culture medium (Zhao et al., 2015), the cells could not survive (Supplementary Figure S2).

### Induction of CiPSLCs From Fish Fibroblasts

In an optimized protocol, every 10^5^ fibroblasts were plated in 30 mm Petri dishes. The optimized induction medium of three stages is shown in Supplementary Table S2. After 24 h (Day 0), the culture medium was replaced by the first stage medium and changed every 2 days. On day 8, epithelial colonies were formed and transferred to the second stage medium. During days 9–10, the concentrations of CHIR99021, Forskolin, and bFGF decreased to 5 μM, 5 μM, and 25 ng/ml, respectively. On day 11, cells were harvested after trypsin digestion, re-inoculated with 1 × 10^5^ cells in each well of 6-well plate, and then transferred to the third stage medium. The ESC-like clones were digested with 0.25% trypsin and cultured in fish iPS cells culture medium (Peng et al., 2019).

### Alkaline Phosphatase Staining

According to the manufacturer’s instructions, alkaline phosphatase staining was performed using the Alkaline phosphatase Detection Kit (Millipore, United States). As previously described, specific methods were carried out (Peng et al., 2019).

### RT-PCR and qRT-PCR

Total RNA was extracted using the phenol/chloroform method. RNA was quantified and transcribed into cDNA using the PrimeScript™ RT reagent Kit with gDNA Eraser (TaKaRa). Then the first-strand cDNAs were used as templates for RT-PCR and qRT-PCR. The PCR primers sequences are listed in Supplementary Table S3. The qRT-PCR amplification protocol was conducted according to the user guide of the SYBR^®^ Green Realtime PCR Master Mix (Selleck) on the ABI7500 Fast system, and amplification conditions were as follows: 50°C for 2 min and 95°C for 10 min, followed by 40 cycles at 95°C for 15 s and 60°C for 1 min. The average threshold cycle (Ct) was calculated for each sample using the 2-ΔΔCt method and normalized to *β*-actin. The specific techniques were all carried out as previously described (Peng et al., 2019).

### Flow Cytometry Analysis

The Kio sperm-like cells were blown down gently by PBS. After being filtered through a 40 μm cell strainer, the cells were incubated with DAPI (Invitrogen) for about 15 min, then examined by flow cytometry (Sysmex-partec, Germany). As previously described, specific methods were carried out (Peng et al., 2020).

### Karyotyping

The cells were cultured in iPS medium with 5.0% (v/v) CO_2_at 28°C. The cells were treated with 0.2 μg/ml colchicine for 3–4 h, digested into single-cell suspension by 0.25% trypsin. The confluent cells were treated with a hypotonic solution of 0.0375 mol/L KCl for 40–60 min, then fixed twice with cold Carnoy’s fixative (methanol: glacial acetic acid = 3:1, v/v) for 40 min each time, then dropped onto the cold slides. The chromosomes were stained with 5% Giemsa solution (Solarbio Inc., United States) for 30 min.

### Immunofluorescence and RNA Probe Hybridization

For immunofluorescence, the cells were fixed in 4% paraformaldehyde for 30 min at room temperature and then treated with 0.3% Triton X-100 for 5 min. Then cells were blocked with 2% BSA for 1 h in PBS. Primary antibodies of the following markers were used: *Oct4* antibody (1:100, GeneTex United States) and *Nanog* antibody (1:100, GeneTex United States). The fluorescently labeled secondary antibodies anti-rabbit IgG (anti-*Oct4* and anti-*Nanog*) were purchased from Jackson Lab (Sacramento, CA, United States). DNA was stained with DAPI. Fluorescence was imaged using a confocal laser scanning microscope (Olympus FV1200, Tokyo, Japan). As previously described, specific methods were carried out (Peng et al., 2019).

For RNA probe hybridization, cells were fixed with 10% formalin for 30 min at room temperature. According to the manufacturer’s instructions, the preamplifier, amplifier, label probe, and chromogenic detection procedures were according to the Advanced Cell Diagnostics RNAscope^®^ 2.0 HD Detection Kit (Hayward, United States) according to the manufacturer’s instructions. The slides were then incubated with anti-ZF-*pou5f1* antibody (520971-C2) and anti-ZF-*Nanog* antibody (520961-C2) overnight at 4°C. The fluorescently labeled secondary antibodies were purchased from Jackson Lab (Sacramento, CA, United States). DNA was stained with DAPI.

### Embryoid Body Formation

The embryoid body (EB) was generated using the method of hanging drop. Briefly, Kio-CiPSLCs were digested into single cells suspension at a density of 10^6^ cells/ml with fibroblast medium, then made a 15 μL hanging drop in 10 cm tissue culture dishes for 48 h. Cell aggregates were collected and transferred to low-adherence containers to allow the development of EBs. As previously described, specific methods were carried out (Peng et al., 2019).

### Fluorescent Dye Labeling and Chimera Formation

The cell preparation and chimera formation were conducted according to Peng et al.'s methods (Peng et al., 2019). The Kio-CiPSLCs were first collected in Diluent C (Sigma, United States) with a density of 10^7^cells/mL. The cells were stained with PHK26 (Sigma, United States) fluorescent dye for 3–5 min 1%BSA was added to terminate the labeling reaction, and the unbound dye was removed by repeated washing with PBS. Chimera formation was evaluated by transplantation PHK26-labeled Kio-CiPSLCs into the mid-blastula stage of zebrafish embryos, as previously described (Peng et al., 2019). The labeled Kio-CiPSLCs were suspended in PBS. Approximately 200–300 donor cells (equal ratio of cells) were injected into the deep cell layer of each embryo. The recipient embryos were incubated in E3 medium at 28.5°C after injection and were monitored under a fluorescent microscope. As previously described, specific methods were carried out (Peng et al., 2019).

### Cell Transplantation in Zebrafish Larvae

The zebrafish larvae (7dph) were anesthetized with 0.02% tricaine and placed on a culture dish. PKH-26 labeled Kio-CiPSLCs were injected directly into the abdominal cavity with 10 μL of cell solution (approximately 1,000 cells). The injected fish were placed in water for 5 h containing 10 ppm tricaine. Ultimately, the fish were bred commonly.

### Sequenommassarray Methylation Analysis

The DNA of samples was treated with sodium bisulfate. Primers to amplify different amplicons in specific regions of Oct4 and Nanog were designed using the Sequenom Epi Designer application (Supplementary Table S3). The treated DNA (5 ng) was used to perform a region-specific PCR reaction incorporating a T7 RNA polymerase sequence. After *in vitro* transcription, RNase A was added to cleave the *in vitro* transcript. The final product was spotted onto a Spectro CHIP array and analyzed using the MassARRAY Compact System.

### Induction of Germ-Like Cells From Kio-CiPSLCs

Methods referred to the protocol of induced pluripotent stem cells to differentiate into germ cell-like cells in mammals (porcine) (Wang et al., 2016). The whole induction scheme was divided into three stages. First, Kio-CiPSLCs (passage 10) were planted in 30 mm Petri dish of 1×10^5^ cell/ml. After 24 h of culture in the incubator, the cells were replaced with the first stage culture medium and treated for 3 days. Then, the culture medium was changed to the second stage and then treated for 8 days. Finally, the culture medium was changed to the third stage and treated for 9 days. Sperm-like cells began to appear under the stimulation of RA and testosterone. For each step, the medium was changed every 2 days.

The first stage culture medium was composed of Dulbecco’s modified Eagle’s medium (DMEM) supplemented with 10% KSR, 1% N2 supplement, 2% B27 supplement, 1%Glu, 1%NEAA, 1%penicillin-streptomycin, 0.055 mM 2-mercaptoethanol, 12 ng/ml bFGF and 20 ng/ml Activin A. The second stage culture medium was composed of DMEM supplemented with 10% KSR, 1% Glu, 1% NEAA, 1% penicillin-streptomycin, 0.055 mM 2-mercaptoethanol, 1000 U/ml LiF, 50 ng/ml BMP4, 50 ng/ml BMP8a, 50 ng/ml SCF and 50 ng/ml EGF. The third stage culture medium was composed of DMEM supplemented with 15% FBS, 1% Glu, 1% NEAA, 1% penicillin-streptomycin, 0.055 mM 2-mercaptoethanol, 2 μM RA, 1 μM testosterone and 5 ng/ml GDNF.

### Scanning Electron Microscopy Analysis

The Kio sperm-like cells were fixed with 2.5% glutaraldehyde (Sigma) for 2 h, then dehydrated in a graded ethanol series and sputter-coated with gold. Samples were then studied at 20 kV (JSM-6360LV, HITACHI, Japan). The specific methods were carried out as previously described (Fu et al., 2021).

### Transmission Electron Microscopy Analysis

The attached Kio sperm-like cells were performed a fixation with 2.5% glutaraldehyde for 2 h at 4°C, post-fixation in 1% osmium tetroxide (Sigma), dehydrated in ethanol series, incubated twice with propylene oxide for 20 min, and embedded in Epon mixture. After embedding, the Epon samples were polymerized for 48 h at 60°C. Ultrathin sections were stained with uranyl acetate and lead citrate. An HT7800 electron microscope (HITACHI, Japan) was used to analyze the specimens at 100 kV. The specific methods were carried out as previously described (Mo et al., 2019).

## Results

### Generation of iPS-Like Cells From Kio Fibroblasts With Small Molecular Compounds

**FIGURE 1:**
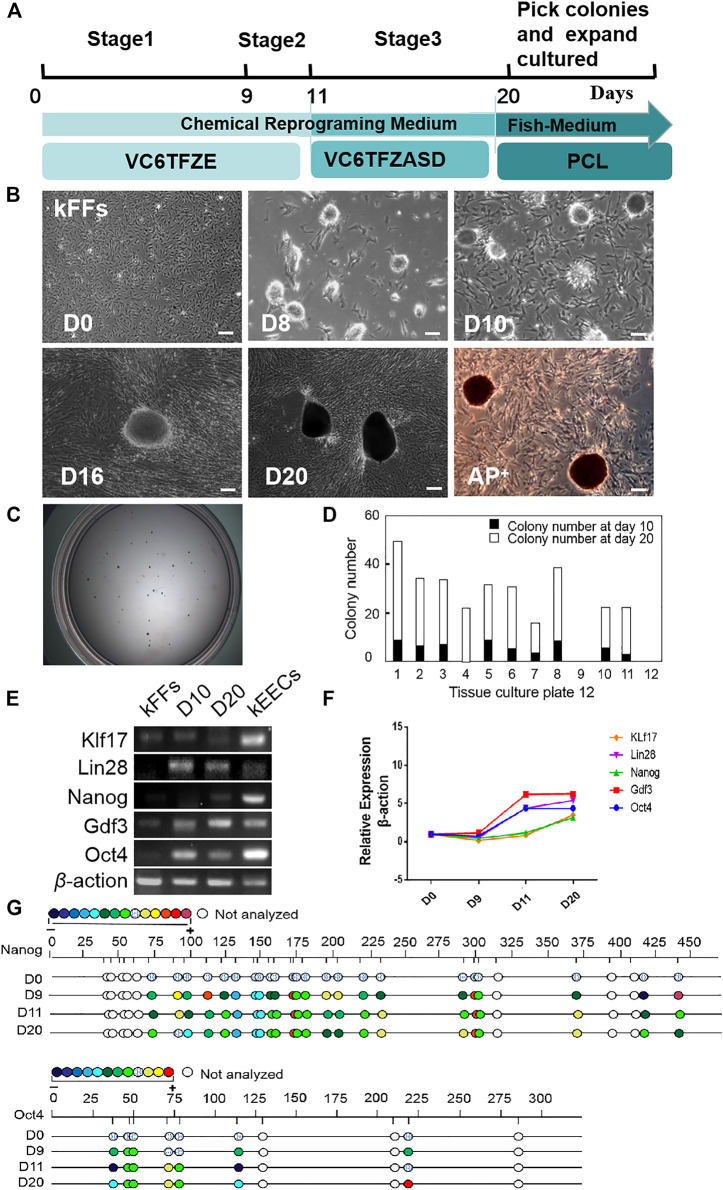
Generation of iPS-like Cells from Kio fin fibroblasts (kFFs) by small-molecule compounds. **(A)** Schematic of the optimized protocol (V, VPA; C, CHIR 99021; 6, 616452; T, tranylcypromine; F, forskolin; Z, DZNep; E, EPZ004777; A, AM580; S,SGC0946; D, 5-aza-dC; P, PD025901; L, LIF). **(B)** The time course and cell morphology of chemical reprogramming of kFFs using the optimized protocol. Dense epithelioid colonies were observed on the eighth day and proliferated with the passage of time. On the 10th day, clear-edged, mindless clones appeared. In the third stage, the number of clones increased significantly, the size of clones increased, and the alkaline phosphatase test showed positive. Scale bars, 100 μm. **(C)** The bright image of colonies in a 30 mm Petri dish at day 20. **(D)** The number of colonies at day 10 (black columns) or day 20 (white columns) after induction. Clones appeared at the end of the first stage of installation, and the number of clones increased significantly at the third stage. The results were obtained from at least three independent experiments. **(E)** RT-PCR analysis of ES cell marker genes in iPS-like cells (day 10 and day 20), kEECs (Early embryonic cells of Kio) and kFFs as control. They have clearly shown that the pluripotent genes were expressed in the second and third stages, which was similar to that in the blastocyst cells of the positive control group but not in the fibroblasts. **(F)** qRT-PCR analysis of ES cell marker genes in iPS-like cells at day 10 and day 20 and kFFs as control. It was clearly shown that the expression of pluripotent marker genes in the induced cells was significantly increased. **(G)** Bisulfite genomic sequencing of the promoter regions of *Oct4* and *Nanog* in iPS-like cells at day 10 and day 20, kFFs at day 0 as control. Different color circles indicate the degree of CpG dinucleotide methylation.

According to the pre-experiments (For details, see Materials and Methods), we have established an induction protocol suitable for kFFs (Figure 1A). As shown in Figure 1B, epithelioid cells aggregation began to appear at the first stage of induction (8 day). After the second stage of induction, the epithelioid cells gathered more closely, and the dense clones were formed. From day 16–20, a large number of clear-edged, dense ESC-like clones appeared, which alkaline phosphatase test showed that they were positive (Figure 1B). The number of clones increased significantly at the third stage, with an average of 20–30 clones per 30 mm Petri dish (Figures 1C,D).

*Oct4, Nanog, Klf17, Lin28,* and *Gdf3* were selected as pluripotent marker genes, which were commonly used in mammals and fish (Takahashi and Yamanaka, 2006; Robles et al., 2011; Liu et al., 2015; Pelliccia et al., 2017). Considering fish early embryonic cells can remain a pluripotent state from zygotic genome activation to the oblong stage (Xiao et al., 2016), and even maintain pluripotency for multiple passages in culture (Fan et al., 2004). Kio early embryonic cells (kEECs) were used as a positive control of pluripotent characteristics.

RT-PCR analysis showed that the pluripotency genes were expressed in both the second and third stages, which was the same as that in kEECs, but not in kFFs (Figure 1E). Fluorescence quantitative PCR results showed that the expression of *Oct4, Lin28,* and *Gdf3* increased significantly, while *Nanog* and *Klf17* increased slightly at Stage 2 (9–11 days). At Stage 3 (days 12–20), the expression of *Nanog, Klf17,* and *Lin28* increased slightly, while *Oct4* and *Gdf3* did not change (Figure 1F). Bisulfite genome sequencing analysis furtherly showed that the promoter regions of *Oct4* and *Nanog* were hypomethylated during induction (Figure 1G). Our data indicated that Kio fibroblasts could be chemically reprogrammed into the pluripotent stem cells, and a cautious adjustment of small molecule compounds played a critical role in ensuring a successful chemical reprogramming in fish.

### Kio-CiPSLCs Derived From kFFs

**FIGURE 2:**
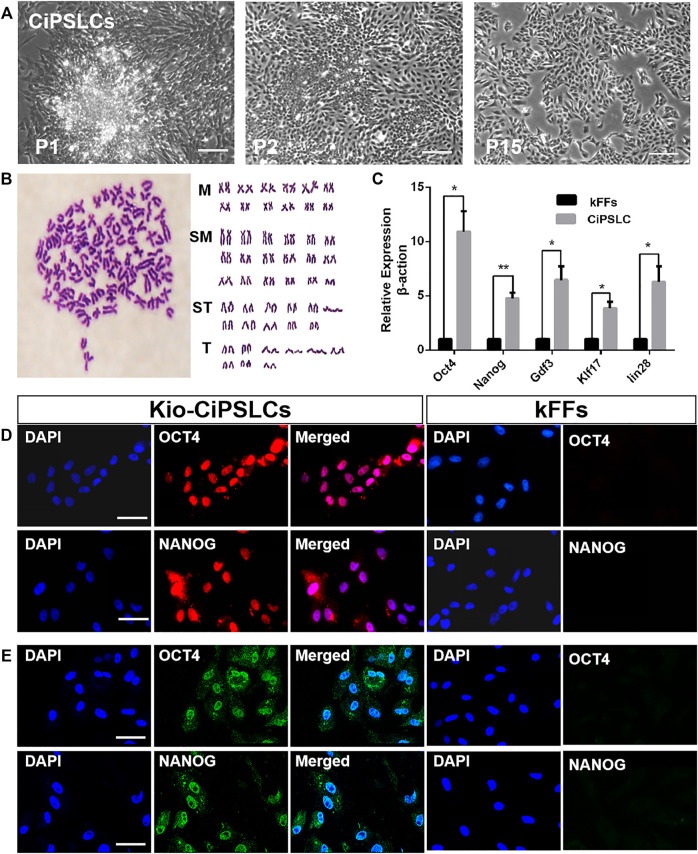
Characteristics of Kio-CiPSLCs derived from kFFs. **(A)** Morphology of Kio-CiPSLCs at passage 1, passage 2 and passage 15. After 15 courses in a fish stem cell culture medium, the stable ES cell-like morphology was maintained. Scale bars, 200 μm. **(B)** Karyotype analysis of Kio-CiPSLCs at passage 20. Kio-CiPSLCs maintained normal karyotype, diploid metaphase of 100 chromosomes as seen in the majority of Kio-CiPSLCs. **(C)** The qRT-PCR analysis of ES marker gene expression in Kio-CiPSLCs and kFFs as control. Clearly shown that the expression of pluripotent genes in Kio-CiPSLCs was significantly higher than that in kFFs. *, *p* < 0.05, **, *p* < 0.01. **(D,E)** RNA probe hybridization **(D)** and immunofluorescence staining **(E)** of Oct4 and N*anog* in Kio-CiPSLCs (passage 15), kFFs as control. Scale bars, 50 μm.

We selected the clones to subculture and then established the iPS cell line from kFFs (Kio-CiPSLCs) (Figure 2A). The Kio-CiPSLCs retained ES cell-like morphology after multiple generations of culture, including round, large nucleoli, and sparse cytoplasm. After 20 generations cultured in fish iPS cells culture medium, the Kio-CiPSLCs maintained a normal karyotype, diploid metaphase of 100 chromosomes as seen in the majority of Kio-CiPSLCs (Figure 2B). Fluorescence quantitative PCR analysis showed that the expression level of pluripotent genes *Oct4, Nanog, Klf17, Lin28*, and *Gdf3* in Kio-CiPSLCs were significantly higher than those of kFFs (Figure 2C). In addition, RNA fluorescence probes hybridization and immunofluorescence staining indicated that both *Oct4* and *Nanog* were expressed in the Kio-CiPSLCs (Figure2D,E). These results reflected, the Kio-CiPSLCs had the molecular expression characteristics of stem cells.

### Differentiation Potential of Kio-CiPSLCs Derived From kFFs

**FIGURE 3:**
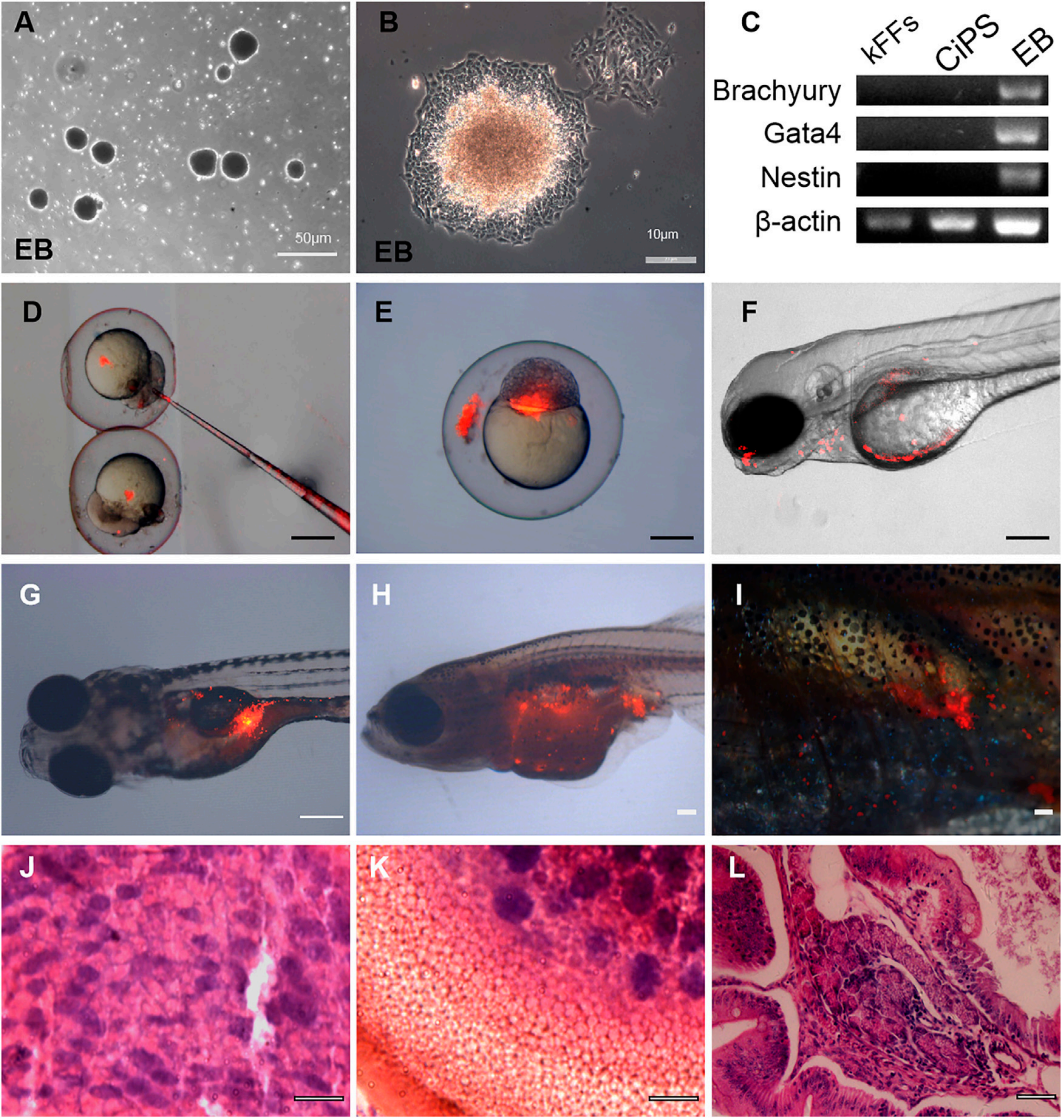
Differentiation potential of Kio-CiPSLCs derived from kFFs. **(A,B)** The embryoid body (EB) formation **(A)** and differentiation **(B)**. **(C)** RT-PCR analysis of ectoderm marker gene (*Nestin*), mesoderm marker gene (*Brachyury*), and endoderm marker gene (*Gata4*) in the three germ layers. **(D–F)** Different distributions of PKH26-labeled donor cells (Kio-CiPSLCs, passage 15, red) in different stages, in which the chimeras were analyzed by microscopy at 5 days post-fertilization **(F)**. Scale bars, 200 μm. **(G–I)** PKH26-labeled Kio-CiPSLCs (passage 15, red) were transplanted with approximately 1,000 cells to host zebrafish, and the teratomas were analyzed after 8 weeks post-injection. Of the 30 injected fish, teratomas were observed in five fish. Scale bars, 200 μm. **(J–L)** Histology of various tissues present in teratomas derived from Kio-CiPSLCs. Neural epithelium (ectoderm) (J); Cartilage (mesoderm) **(K)**; Gut-like epithelium (endoderm) **(L)**. Scale bars, 50 μm.

To test the differentiation ability of Kio-CiPSLCs *in vitro*, Kio-CiPSLCs were cultured in uncoated plastic Petri dishes to form embryoid bodies (Figure 3A). After continuing culture in the tissue Petri dish, the embryoid bodies were attached to the Petri dish’s bottom and began to differentiate (Figure 3B). RT-PCR analysis showed that endoderm (*Gata4*), mesoderm (*Brachyury*), and ectoderm (*Nestin*) marker genes were all expressed in the embryoid body (Figure 3C). These results showed that Kio-CiPSLCs could differentiate into three germ layer cells *in vitro*.

We further tested the developmental potential of Kio-CiPSLCs *in vivo*. The Kio-CiPSLCs were collected and stained with PKH26 reagent, then injected into zebrafish embryos at the blastocyst stage (8–10 h after fertilization). We found that the tested Kio-CiPSLCs were successfully integrated into zebrafish organisms, and some of the integration occurred in the head and abdomen (Figures 3D–F). Kio-CiPSLCs integration in gonadal tissue was observed in a small number of individuals, but it was uncertain whether there was germline transmission (Figures 3G–I).

We also examined the pluripotency of Kio-CiPSLCs via the formation of teratoma. The Kio-CiPSLCs were injected into juvenile zebrafish about 7 days after hatching. Finally, well-differentiated teratomas were formed in five zebrafish. Histological examination showed that the teratomas contained all three layers of germ tissue, such as nerve tissue, cartilage, and columnar epithelium (Figure 3J–L). In summary, our results showed that the Kio-CiPSLCs obtained by pure chemical reprogramming were pluripotent and fully reprogrammed.

### Kio-CiPSLCs Differentiation Into Germ Cell-Like Cells *in Vitro*

**FIGURE 4:**
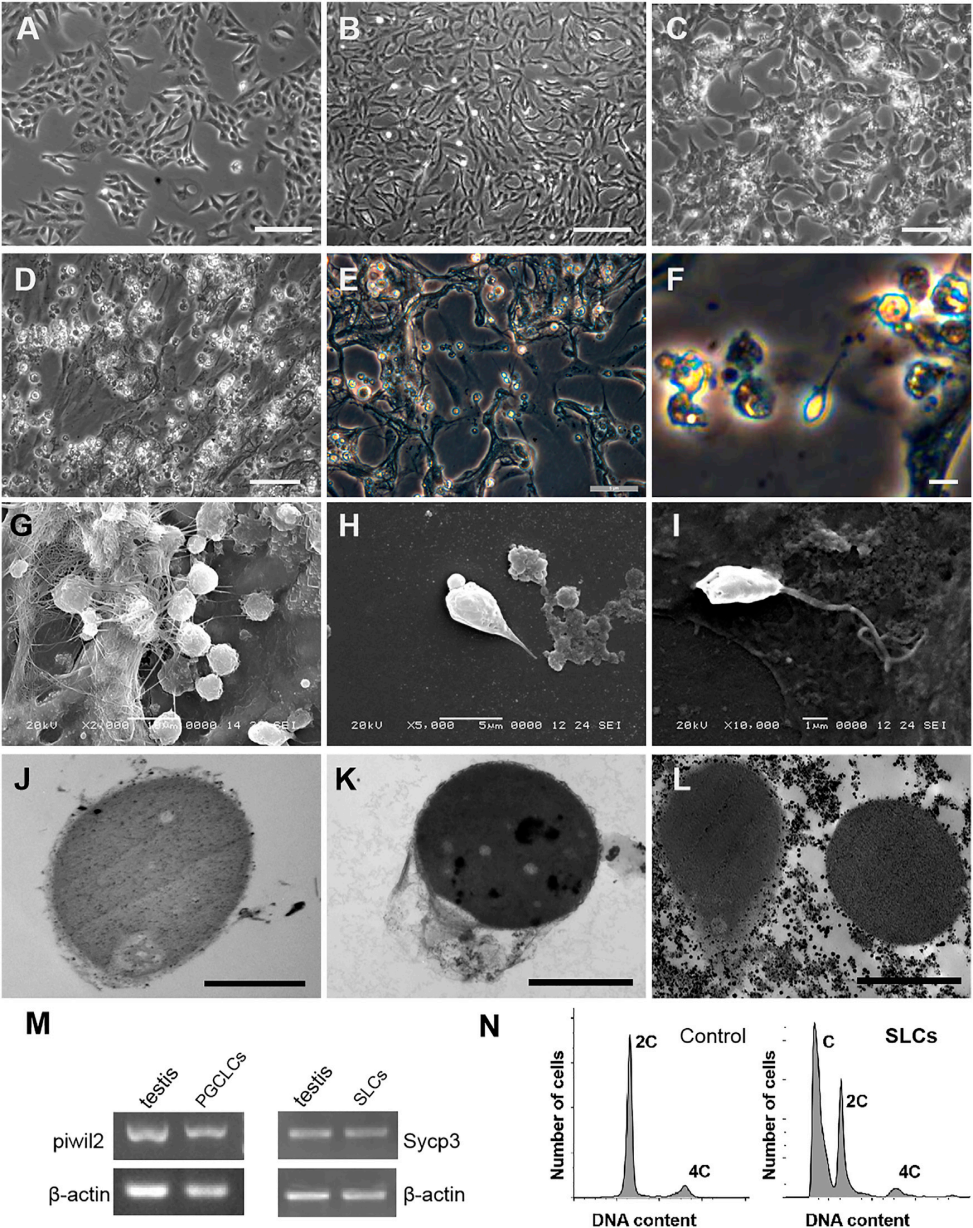
Generation of germ cell-like cells from Kio-CiPSLCs. **(A)** Morphology of Kio-CiPSLCs. **(B–F)** Morphology of the induced cells. On day 3, the cells became long and narrow **(B)**; On day 7, the cells enlarged and returned to a shape similar to that of stem cells **(C)**; On day 11, the cells became smaller and a large number of small spherical cells appeared **(D)**; On the day 20, sperm-like cells (SLCs) with long tails appeared **(E,F)**. **(G–I)** Morphology of the induced cells (day 20) was observed by scanning electron microscopy. A large number of sperm-like cells with small spheres and slender tails were observed. **(J–L)** Morphology of the induced cells (day 20) was observed by transmission electron microscopy. It has typical sperm cell morphology, such as nuclear condensation, cytoplasmic loss, and microtubule structure similar to sperm tail. **(M)** RT-PCR analysis of *Piwil2* and *Sycp3* expression levels in day 10 and day 18-induced cells. The cells form Kio testis as control. **(N)** Flow cytometry analysis of the day 20-dinduced cells. kFFs as control. A haploid peak appeared, indicating that Kio-SLCs have completed meiosis. **(A–D)** Scale bars, 20 μm; **(E–L)** Scale bars, 5 μm.

The scheme of induce Kio-CiPSLCs to differentiate into germ cell-like cells was referred to Wang et al. (2016) (For details, see Materials and Methods). The Kio-CiPSLCs retained ES cell-like morphology after multiple generations of culture (Figure 4A). After the first stage of treatment (days 1–3), the morphology of cells changed obviously, and the cells became long and narrow (Figure 4B). On the seventh day of the second stage, the cells enlarged and returned to a round shape similar to that of stem cells (Figure 4C). At the end of the second stage (day 11), the cells became smaller and a large number of small spherical cells appeared (Figure 4D). Sperm-like cells with tails appeared at the end of the third stage (day 20) (Figures 4E,F). The morphological examination of the cells at the end of the third stage (20th day) was performed by electron microscope. We observed a large number of small globular cells attached to the underlying fibroblasts (Figure 4G). Sperm-like cells with oval heads and tails could also be observed (Figures 4H,I). Ultrastructures observation showed that the sperm-like cells had a large nuclear-cytoplasmic ratio and a very high electron density in the nuclear region. However, we did not find the shaft of spermatozoal flagellum (Figures 4J–L).

The cells of Stage2 (10th day) and Stage3 (18th day) were selected for molecular identification. The testis of Kio was used as a positive control. RT-PCR showed that the germ cell marker gene *Piwil2* could be detected in the 10th-day cells and the meiotic marker gene *Sycp3* could be detected in the 18th-day cells (Figure 4M). Flow cytometry analysis showed the 18th-day cells had haploid and double peaks, which further proved that part of the cells has completed meiosis (Figure 4N).

## Discussion

In this study, we generated iPS-like cells from caudal fin fibroblasts of Kio with a pure chemical reprogramming method for the first time. The characteristics of iPS-like cells include colony morphology, expression of pluripotent marker genes, formation of embryoid bodies and teratoma, and the potential to differentiate into germ cell-like cells *in vitro*. Our results showed that the process of cell reprogramming induced by small molecular compounds was relatively conservative in different species. These findings suggested that the pure chemical reprogramming method was a promising strategy and may be extended to more species.

It has been reported that there are significant differences in stem cells between fish and mammals. For example, different pluripotent marker genes, different culture conditions, and so on (Robles et al., 2011; Ho et al., 2014; Hong et al., 2014). Referring to Deng and his team’s chemical reprogramming manual in mice (Hou et al., 2013; Zhao et al., 2015; Ye et al., 2016). During the induction of fish pluripotent stem cells, we found that fish cells were more sensitive to small molecules. Therefore, the treatment of a low concentration of small molecules is the key to success. Meanwhile, compared with mammals, clone clusters appeared earlier in the process of fish cell induction. In fish, clones begin to appear in the first stage of installation, but they do not appear until the second stage of induction in mice (Zhao et al., 2015; Ye et al., 2016). In addition, long-term culture and preservation of pluripotency of CiPSLCs in fish was also a challenge. When cultured in mammalian iPS medium, Kio-CiPSLCs could not survive. With reference to the composition of fish embryonic stem cell culture medium, combined with our previous experience in culturing zebrafish pluripotent stem cells (Peng et al., 2019), we optimized the culture medium for Kio-CiPSLCs, and the Kio-CiPSLCs could be cultured for a long time. Finally, we obtained the iPS-like cells by adjusting the concentration of small molecular compounds and treatment time. Our research showed that although the pure chemical reprogramming process was relatively conservative in mammalian and non-mammal species, a cautious adjustment of these small chemical molecules was the key to the success of the reprogramming of fish cells.

Recently, mouse ESCs/iPSCs have been used to produce sperm-like cells or mature oocytes *in vitro* (Hayashi and Saitou, 2014; Saitou and Miyauchi, 2016; Bharti et al., 2020). In fish, there are also studies on the use of zebrafish testicular proliferative cells to produce functional sperm from self-renewing spermatogonial stem cells (SSCs) (Kawasaki et al., 2016). In this study, the morphology and expression of meiotic marker genes of germ cell-like cells directly differentiated from Kio-CiPSLCs were similar to those of sperm-like cells induced in fish and mammals (Hong et al., 2004; Kawasaki et al., 2016; Wang et al., 2016; Zhou et al., 2016). During the spermatogenesis of common carp (*Cyprinus carpio*), the secondary spermatocytes also formed the typical circular condensed nucleus and spermatids were further packed with characteristic of higher nucleo-cytoplasmic ratios (Zhu et al., 2021). Compared with the real iPSCs, the chemically induced pluripotent stem cell-like cells we induced can produce most of the cell types of adult fish but have not been able to produce functional gametes. It is absolutely essential to induce efficient germ-like cells from pluripotent stem cells *in vitro*. A new 3D culture system may be a way to solve the problem of large-scale cultivation. There are attempts to improve the culture efficiency of germ-like cells by using 3D culture system in mammals (Ganjibakhsh et al., 2019; Wang et al., 2019). Using 3D culture system may be a helpful way to further optimize the induction scheme for the directional differentiation of iPS-like cells into primordial germ cells and finally obtain functional sperm in fish.

## Data Availability

The original contributions presented in the study are included in the article/Supplementary Materials, further inquiries can be directed to the corresponding authors.
