# Supplementary material for: Fish Pluripotent Stem-Like Cell Line Induced by Small-Molecule Compounds From Caudal Fin and its Developmental Potentiality
Source: Front Cell Dev Biol. 2022 Jan 20;9:817779. doi: 10.3389/fcell.2021.817779 (PMC8811452; doi:10.3389/fcell.2021.817779)
Supplement: Supplementary file 1 [file DataSheet1.PDF]

Table S1. Information of the small molecule compounds.

Full Name (Abbr.)	Source
Valproic acid sodium salt (VPA, V)	Sigma
Tranylcypromine (Tranyl, T)	Sigma
CHIR99021 (CHIR, C)	BioVision
AM580 (A)	Tocris
Forskolin (FSK, F)	Selleck
Repsox (616452)	Selleck
EPZ004777 (EPZ, E)	Selleck
SGC0946 (SGC, S)	Selleck
3-deazaneplanocin A (DZNep,Z)	Selleck
5-aza-dC (D)	Enzo
2-mercaptoethanol	Sigma
leukemia inhibitory factor (LIF)	Miliproole

Table S2.Induction medium of three stages (Knockout DMEM)

Medium composition	First stage (Day 1-8)	Second stage (Day 9-10)	Third stage (Day 11-19)
KOSR	10%	10%	10%
FBS	10%	10%	10%
Carp serum	2.5%	2.5%	2.5%
GlutaMAXTM-I	1%	1%	1%
NEAA	1%	1%	1%
Penicillin-Streptomycin	1%	1%	1%
bFGF	100 ng/ml	25 ng/ml	25 ng/ml
VPA	0.25 mM	0.25 mM	0.25 mM
CHIR99021	10 µM	5 µM	5 µM
Repsox	5 µM	5 µM	5 µM
Tranylcypromine	2.5 µM	2.5 µM	2.5 µM

Forskolin	25 μM	5 μM	5 μM
AM580	0.025 μM	0.025μM	0.025 μM
EPZ004777	2.5μM	2.5μM	/
DZNep	/	/	0.025μM
5-aza-dC	/	/	0.25μM
SGC0946	/	/	2.5 μM

Table S3. Primer sequences in this paper

Gene	Name	Sequence（5’-3’）
pluripotent marker genes	Oct4	F-GGAGCTACTACTGATCGCCG
		R-CGAGGCTTGAGGGTACATGG
	Nanog	F-TTCTACCCCAGCTACCCAC
		R-TCCTTTCGTTGTCCACTG
	Gdf3	F-GCTGCGTTCCCATCAAACTG
		R-ATCTGCATCCACACTCGTCC
	Tdgf1	F-TCGTAGGACTGACCGGAGTT
		R-TCCACATCTGCAGTACGAGC
	Klf17	F-GGAAGGTTGTGGCTGGAAGT
		R-TGATCTGAGCGGGAGAAAGC
	β-actin	F-CGTCTTCCCCTCCATCGTTG
		R-ATCTTCTCCATGTCGTCCCA
three dermal layer related genes	Brachyury	F-TGACCAAATATGTGCATT
		R-ATATCCAAACTGCGAGG
	Gata4	F-TGAGCCCTAATATCGGAGCCT
		R-CTCTCCTTCTGCATTGCGTCT
	Nestin	F-ATGCTGGAGAAACATGCCAT
		R-CATCAGGCTGTCAATCTGCT
germ cell marker genes	Piwil2	F-CCCAACGCTGTAGTGATTCTCG

	Sycp3	R-TGAGACCGCTTCGTTATTGCT<br>F-GGTTACTCTTCATACAGCGAGA<br>R-TCATTTTCTGGTTTTGCACGA
methylation analysis of related genes	Oct4	F-aggaagagagTTTTGTTAATTGATTTGGAGGTTTT<br>R-cagtaatacgactcactatagggagaaggctACTTATTTATCCTCTCCAACTCATCC
	Nanog	F-aggaagagagGGGTTTAGTTTTATAAGAGAGGAAGAGT<br>R-cagtaatacgactcactatagggagaaggctTCCATCAAATCATAACAAAAAATCA

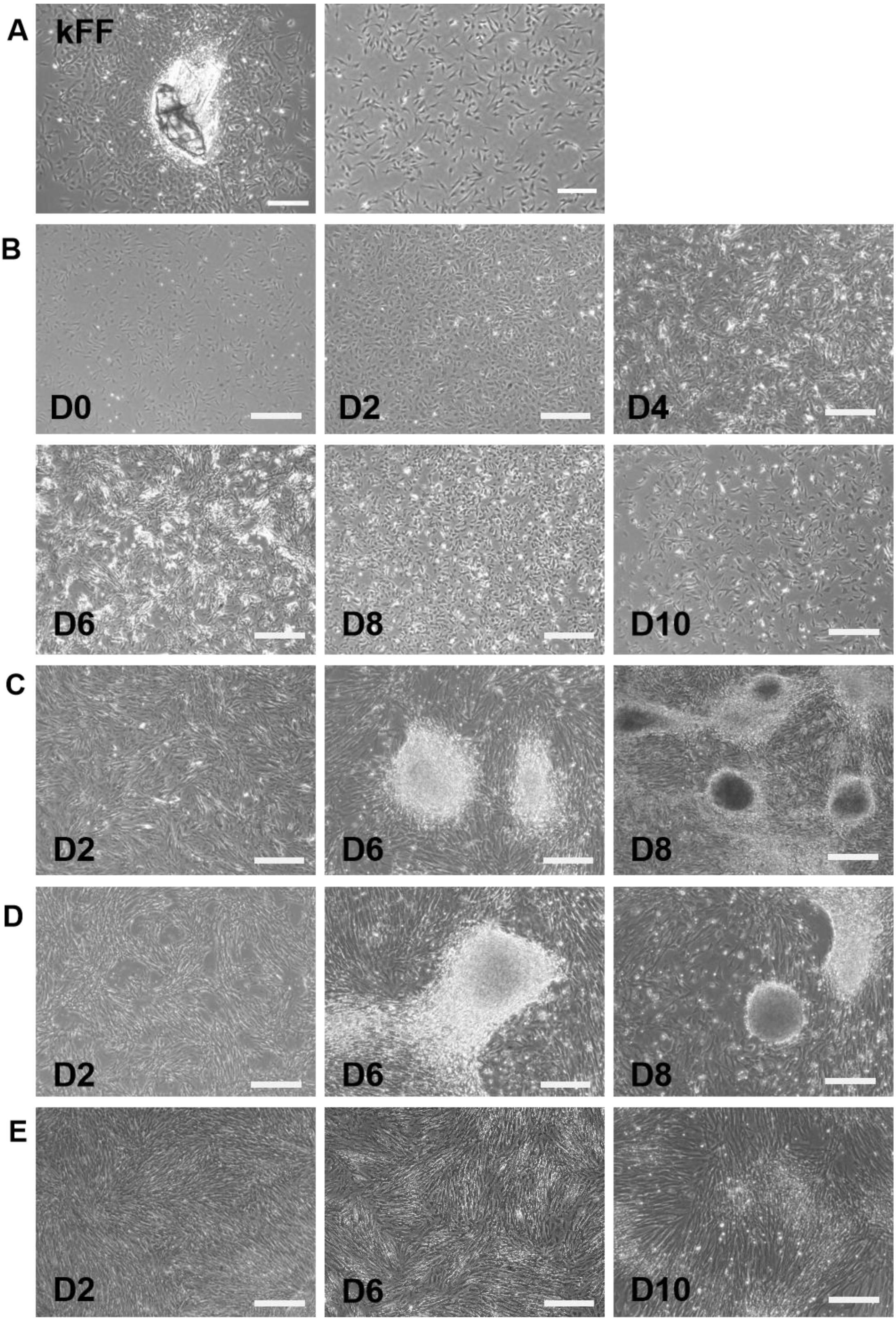

Figure S1 The time course and cell morphology of chemical reprogramming of kFFs.

(A) The morphology of primary cell culture from caudal fin of Kio.

(B) The time course and cell morphology of kFFs that treated with original concentration of small molecular compounds. A large number of cells lost their vitality after 10 days of treatment.
(C) The time course and cell morphology of kFFs that treated with 50% of original concentration. Dense epithelioid colonies were observed on the 8th day and proliferated with the passage of time.
(D) The time course and cell morphology of kFFs that treated with 25% of original concentration. Epithelioid colonies were also observed on the 8th day. However, these colonies had flatter, loose cell morphology, and did not form dense epithelioid colonies in the subsequent induction process.
(E) Treated with 20% of original concentration. No epithelioid colonies were observed during the whole induction process. Scale bars, 200μm.

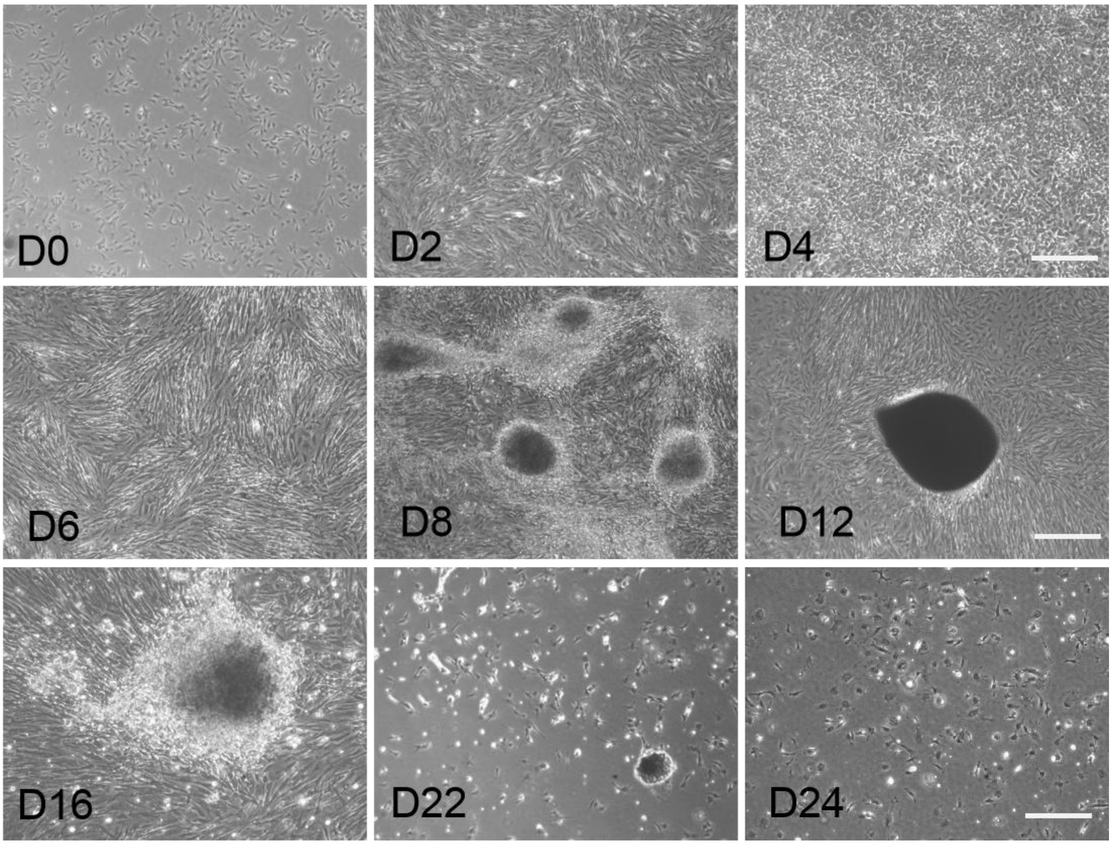

Figure S2 The time course and cell morphology of chemical reprogramming of kFFs using diluted small molecular compounds.
In the second stage (day 9-11) induced by low concentration of small molecules, clear-edged and dense clones appeared. However, after switching to the third stage culture medium, the cells could not survive again. Scale bars, 200 μm.
